# Posterior reversible encephalopathy syndrome in a survivor of valproate-induced acute liver failure: a case report

**DOI:** 10.1186/1752-1947-7-144

**Published:** 2013-05-31

**Authors:** Sachith Mettananda, Asvini D Fernando, Nimasari Ginige

**Affiliations:** 1 Department of Paediatrics, Faculty of Medicine, University of Kelaniya, P.O. Box 6, Ragama, Sri Lanka; 2 Colombo North Teaching Hospital, Ragama, Sri Lanka

**Keywords:** Acute liver failure, Carnitine, Reversible encephalopathy, Valproate

## Abstract

**Introduction:**

Posterior reversible encephalopathy syndrome is an extremely rare radiological diagnosis that has not been reported previously in association with acute liver failure.

**Case presentation:**

A 6-year-old Sri Lankan girl developed acute liver failure with severe hepatic encephalopathy due to sodium valproate. She was successfully treated medically with N-acetylcysteine and L-carnitine. During recovery she again developed features of encephalopathy and had repeated convulsions associated with moderate hypertension. The diagnosis of posterior reversible encephalopathy syndrome was made on clinical and radiological grounds and she showed a gradual improvement with control of blood pressure.

**Conclusions:**

This report adds to the evidence behind treatment of valproate-induced acute liver failure with N-acetylcysteine and L-carnitine and illustrates a rare but interesting association between acute liver failure and posterior reversible encephalopathy syndrome.

## Introduction

Hepatotoxicity is a well-recognized complication of sodium valproate therapy and when progressed to acute liver failure (ALF) carries a lethal prognosis with a transplant-free survival below 25% [1]. Medical treatment of ALF is currently imperfect even in the best of centers and evidence-based options are limited [2].

Posterior reversible encephalopathy syndrome (PRES) is an extremely rare radiological diagnosis especially in children. We report a pediatric patient with valproate-induced ALF successfully treated with N-acetylcysteine (NAC) and L-carnitine who developed PRES during recovery.

## Case presentation

A 6-year-old Sri Lankan girl who was on sodium valproate 20mg/kg per day for 3 weeks for complex partial seizures presented with fever, anorexia and vomiting of 2 days’ duration. She was previously well except for the seizures and was developing normally. She weighed 27kg (97th centile), had a generalized erythematous rash and mild hepatomegaly. The rest of the physical examination was normal. Full blood count and liver function tests done on admission were normal (Table 1).

**Table 1:** Results of biochemical investigations at different time points during hospital stay

	**Investigation results on admission**	**Investigation results at diagnosis of valproate-induced hepatotoxicity**	**Investigation results during ventilation for hepatic encephalopathy**	**Investigation results at diagnosis of posterior reversible encephalopathy syndrome**
Hemoglobin (g/dL)	13.0	13.2	12.8	10.0
White cell count (/L)	7.6 × 10^9^	28.0 × 10^9^	33 × 10^9^	19 × 10^9^
Neutrophils (%)	50%	44%	38%	40%
Lymphocytes (%)	48%	46%	48%	48%
Eosinophils (%)	2%	10%	14%	12%
Platelet count (/L)	157 × 10^9^	411 × 10^9^	409 × 10^9^	544 × 10^9^
Alanine aminotransferase (U/L)	18	625	1026	702
Aspartate aminotransferase (U/L)	43	1199	2768	1024
Serum bilirubin (g/dL)		0.5	13.0	9.9
Serum albumin (g/dL)		2.9	2.3	3.2
Serum globulin (g/dL)		1.9	2.5	3.6
International normalized ratio		1.48	3.25	1.3
Serum alkaline phosphatase (U/L)		1839	1272	1043

She continued to be ill and have high fever without a focus for the next 7 days. She developed cracking of the lips and her liver enlargement was progressive to be palpable 6cm below the costal margin. She was normotensive, conscious and rational. The results of investigation done on day 7 after admission revealed a rapid rise in hepatic transaminases (Table 1). Erythrocyte sedimentation rate was 15mm/hour and C-reactive protein was 12mg/dL. Renal function tests, blood sugar and blood pH were normal. An ultrasound scan of her abdomen revealed hepatomegaly with thickened gallbladder.

Valproate-induced hepatotoxicity was suspected and the drug was discontinued immediately. The patient was started on NAC infusion. Despite treatment with NAC, she deteriorated over the next 24 hours and progressed to hepatic encephalopathy and needed elective intubation and ventilation in an Intensive Care Unit (ICU). She was deeply comatosed over the next 2 days with severe encephalopathy. Hepatic transaminases continued to rise, serum bilirubin increased to 13mg/dL and international normalized ratio rose to 3.25 (Table 1). During this period NAC infusion was continued, supportive therapy with intravenous hydration, fresh frozen plasma, intravenous mannitol, broad spectrum antibiotics, phosphate enema, lactulose and routine monitoring were continued. Intravenous L-carnitine 100mg/kg loading dose was given and continued at 50mg/kg twice a day for 2 days. Due to the limited availability of the drug in the country the treatment with L-carnitine was continued with its oral preparation thereafter. After 4 days in the ICU she showed improvement in her level of consciousness and the liver enzymes started to drop. Two days later she was extubated and had a Glasgow Coma Scale (GCS) of 15.

**Figure 1:**
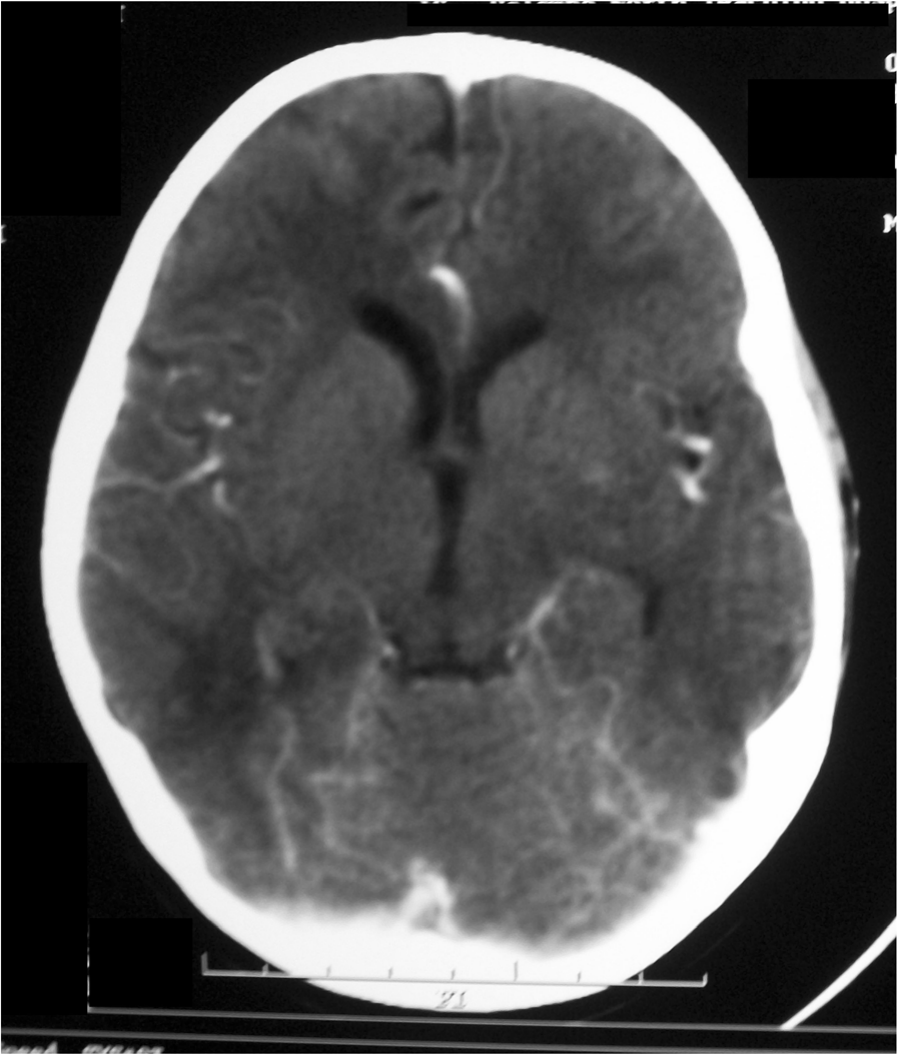
Contrast-enhanced computed tomography scan of brain showing bilateral symmetrical paramedian hypodensities involving cortical and subcortical white matter seen in posterior parietal regions suggestive of posterior reversible encephalopathy syndrome.

On the following day her blood pressure increased abruptly to 140/100mmHg (above the 99th centile). She developed several multifocal clonic convulsions with alternating involvement of both upper limbs and her consciousness level deteriorated again. She was unable to complain of headache or visual disturbances. The results of biochemical investigations including electrolytes, calcium, and sugar were normal. Liver transaminases continued to drop (Table 1). Due to recurrent convulsions a contrast-enhanced computed tomography (CT) scan of her brain was performed. The CT scan showed multiple hypodense areas involving cortical and subcortical white matter in bilateral posterior parietal regions (Figure 1). PRES was diagnosed and her blood pressure was controlled using intravenous hydralazine and magnesium sulfate. Seizures were managed with intravenous midazolam. She was reintubated, ventilated and NAC and L-carnitine were omitted.

Blood pressure normalized with therapy, GCS improved rapidly and she was extubated after 2 days of ventilation. She did not develop further convulsions and was discharged home after 5 days. During review after 2 weeks and monthly reviews thereafter she continued to be free of seizures, without any neurological impairment, normotensive and had normal liver functions even after 6 months.

## Discussion

Drug-induced liver injury (DILI) is a diagnostic and therapeutic challenge [3]. The true incidence of DILI remains unknown but an incidence of up to 14 cases per 100,000 has been reported [1-3]. DILI is due to predictable dose-dependent toxicity or idiosyncratic reactions as in the case with sodium valproate. A minority (25% to 30%) of patients with DILI present with symptoms suggestive of immunoallergic drug reaction with fever, rash and eosinophilia [3]. Our patient had all these features and probably had this type of reaction to valproate.

ALF in children is difficult to manage and carries a high risk of mortality even in countries where liver transplantation is widely available. Mortality from ALF due to drugs (excluding paracetamol) is more than 75% [1]. In Sri Lanka, only a few cadaveric liver transplants have been performed and experience is limited on live-related or pediatric transplant. Hence our options were restricted to meticulous supportive care and medical treatment modalities.

NAC has a proven benefit in the treatment of ALF due to paracetamol overdose [2]. In a prospective double blind trial in the United States of America intravenous NAC infusion for 72 hours improved the transplant-free survival in patients with early stage non-paracetamol-related ALF [4]. The effectiveness of NAC is attributed to anti-oxidant and anti-inflammatory mechanisms as well as to its action on modulating the intrinsic pathway of apoptosis [1-5]. Our patient received NAC early in the disease process before developing encephalopathy. Although it could not prevent progression to encephalopathy it probably helped in ultimate survival without transplantation or long-term sequel.

In a few previous case reports intravenous L-carnitine had beneficial effects in management of acute poisoning, idiosyncratic hepatotoxicity and hyperammonemic encephalopathy due to valproate [6-10]. It has been hypothesized that valproate therapy may induce a carnitine deficiency [10] and some data suggest valproate-induced hepatotoxicity may be promoted by pre-existing carnitine deficiency or by deficiency induced by valproate per se [6]. There was no definite evidence for a suitable dose of L-carnitine; hence we used a loading dose of 100mg/kg followed by 50mg/kg twice a day.

The most striking feature illustrated in this case report is the development of PRES during recovery which has not been reported previously to the best of our knowledge. PRES is a poorly understood entity characterized by headache, seizures, encephalopathy, visual disturbances and radiologic findings of focal reversible vasogenic edema [11]. In this patient PRES was diagnosed due to the reappearance of seizures and features of encephalopathy despite improvement in liver transaminase levels and the characteristic features in the contrast-enhanced CT scan.

Recognized causes of PRES include acute hypertension, pre-eclampsia, renal disease, sepsis, autoimmune disease and exposure to immunosuppressants [11]. It has been reported in association with glomerulonephritis [12], treatment of leukemia [13], liver transplantation [14], steroid treatment [15] and cyclical vomiting [16]. A recent case report described an association between PRES and chronic liver failure and the authors suggested hyperammonemia as a possible etiological factor [17]. The causal relationship between PRES and hypertension is controversial; many believe hypertension to be a cause but there is some evidence to suggest that it is a secondary effect [13]. In our patient an acute rise in blood pressure was detected with the onset of other features of PRES and it was transient. Notably we do not have an alternative explanation for the hypertension. This makes us believe it to be a feature or a secondary effect of PRES rather than the cause. However, the symptoms and signs of PRES disappeared with treatment of hypertension as is the case in many other reports [12,18]. This indicates that hypertension may have an effect in the progression of the disease.

Another explanation would be that PRES is a variant of cerebral edema which is a common complication of ALF. However, in this patient the clinical picture of PRES developed during recovery from ALF. Further, both NAC and L-carnitine need to be considered a cause or causes of PRES although it has not been reported previously, which may be due to the infrequent usage of both drugs. Both drugs were omitted after development of PRES.

## Conclusion

In conclusion this report adds to the evidence behind treatment of valproate-induced ALF with NAC and L-carnitine and illustrates a rare association between ALF and PRES.

## Consent

Written informed consent was obtained from the parents of the patient for publication of this case report and accompanying images. A copy of the written consent is available for review by the Editor-in-Chief of this journal.

## Competing interest

The authors declare that they have no competing interests.

## Authors’ contributions

All authors contributed in patient management, writing up the manuscript and approval of the final manuscript.
